# Adaptive Windowing Framework for Surface Electromyogram-Based Pattern Recognition System for Transradial Amputees

**DOI:** 10.3390/s18082402

**Published:** 2018-07-24

**Authors:** Ali H. Al-Timemy, Guido Bugmann, Javier Escudero

**Affiliations:** 1 Biomedical Engineering Department, Al-Khwarizmi College of Engineering, University of Baghdad, Baghdad 47146, Iraq; 2 Centre for Robotics and Neural Systems (CRNS), Cognitive Institute, Plymouth University, Plymouth PL4 8AA, UK; g.bugmann@plymouth.ac.uk; 3 School of Engineering, Institute for Digital Communications, The University of Edinburgh, Alexander Graham Bell Building, Edinburgh EH9 3FG, UK; javier.escudero@ed.ac.uk

**Keywords:** adaptive windowing, classification, Linear Discriminant Analysis, pattern recognition, surface electromyogram (sEMG), Time-Domain Power Spectral Descriptors, transradial amputees

## Abstract

Electromyogram (EMG)-based Pattern Recognition (PR) systems for upper-limb prosthesis control provide promising ways to enable an intuitive control of the prostheses with multiple degrees of freedom and fast reaction times. However, the lack of robustness of the PR systems may limit their usability. In this paper, a novel adaptive time windowing framework is proposed to enhance the performance of the PR systems by focusing on their windowing and classification steps. The proposed framework estimates the output probabilities of each class and outputs a movement only if a decision with a probability above a certain threshold is achieved. Otherwise (i.e., all probability values are below the threshold), the window size of the EMG signal increases. We demonstrate our framework utilizing EMG datasets collected from nine transradial amputees who performed nine movement classes with Time Domain Power Spectral Descriptors (TD-PSD), Wavelet and Time Domain (TD) feature extraction (FE) methods and a Linear Discriminant Analysis (LDA) classifier. Nonetheless, the concept can be applied to other types of features and classifiers. In addition, the proposed framework is validated with different movement and EMG channel combinations. The results indicate that the proposed framework works well with different FE methods and movement/channel combinations with classification error rates of approximately 13% with TD-PSD FE. Thus, we expect our proposed framework to be a straightforward, yet important, step towards the improvement of the control methods for upper-limb prostheses.

## 1. Introduction

In Iraq, successive wars and violent terrorist attacks have resulted in a large number of people losing their upper limbs. Despite the large number of amputees there and in the rest of the world, they are yet to be provided with hand prostheses that meet their expectations, such as high dexterity and easy controllability. Poor functionality and limited usability are some of the main problems of the existing prostheses. 

Pattern Recognition (PR) based EMG control may offer a promising approach to control the advanced dexterous hands such as the i-Limb hand or Michelangelo hand [1] with the ability to intuitively control a large number of movements with fast reaction times, unlike the conventional EMG control [2,3]. In spite of the recent advances in the field of EMG-based PR systems, where remarkable achievements have been reached in terms of classification accuracy and the ability to control large number of movements, PR systems have been recently deployed in commercial prostheses [4]. However, their use is still very limited. Performance degradation in real life situations due to changing arm posture [5], force variation [3] and signal non-stationarity [6] are some of the barriers which may limit the clinical implementation of PR systems. This is despite a number of studies on processing methods having been tested to recognize hand movements including, for example, nonlinear measures based on recurrence plot to assess the hidden dynamical characteristics of sEMG [7] or modelling sEMG signals of hand manipulations with expectation maximization (EM) algorithms [8]. 

To improve the classification performance, robustness and usability of PR systems, as well as to overcome the limitations of the conventional EMG control, researchers have proposed many enhanced PR systems. Examples of PR system enhancements included post-processing techniques such as Majority Voting (MV) [9], Bayesian Fusion (BF) [10] and self-correcting systems [11]. 

Other approaches to PR enhancement included rejection-based methods which were also proposed to improve the usability of PR systems [12,13,14]. Scheme et al. [12] proposed an extended Linear Discriminant Analysis (LDA) capable of movement rejection. Fitt’s law testing framework was utilized to evaluate the proposed rejection scheme on intact-limbed and amputee subjects. If the system is not robust enough, it may fail to produce the correct decision as a result of outputs with low classifier probabilities; for instance, when the amputee changes the movement a lot or very quickly. In [11], a novel post-processing technique using LDA with maximum likelihood and an artificial neural network was proposed and tested with EMGs from intact-limbed and amputee subjects. However, the proposed technique has numerous parameters that need to be adjusted for each amputee in order to achieve maximal performance.

Other approaches of PR systems enhancements include the adaptation of classifiers [13,15,16,17] and the ensemble of multiple classifiers [13,18,19], which are methods based on the improvement at the classifier level. An unsupervised adaptation strategy of LDA based on probability weighting and cycle substitution was proposed in [20] and tested with EMG signals acquired from five able-bodied subjects for ten classes of movement. In contrast, in [13], a classification scheme was developed based on boosting and random forest classifiers where a threshold was used to adjust the classification and the rejection of the samples belonging to some untrained classes. The approach was validated on EMGs for seven movements from six intact-bodied subjects. This approach is a good example of utilizing two techniques, ensemble boosting and rejection methods, to enhance the performance PR systems.

PR system enhancements may help to improve the existing prostheses and to make them available for amputees’ use. In the real world, the amputee may prefer a prosthesis that can achieve minimal errors with some delay for only suspicious (low confidence) decisions rather than a system which is fast but makes a lot of errors. For instance, a system with 90% accuracy may have one wrong movement in every ten movements which may be catastrophic for the amputee in real life such as dropping off a hot cup of coffee or tea on the amputee which eventually may result in secondary prostheses rejection [21]. Furthermore, the statistical properties of the EMG recorded in real-world situations may be different from those acquired in laboratory conditions [22]. Hence, rigid PR systems will face additional difficulties in their translation to clinical use by patients. This emphasizes the need for systems that have some degree of adaptiveness to facilitate reducing the gap between research and industry [2,22].

Englehart and Hudgins [23] showed that there is a trade-off between the classification accuracy and time window size for PR systems based on the classification of overlapped windows. Therefore, the performance of PR systems can be improved simply by increasing the size of the time window for the misclassified decisions, something that will reduce the variance or variability within the features of the EMG [24]. Most PR systems adopt a fixed and relatively short window size [25,26,27] allowing for acceptable response time delays [28]. However, this entails a risk of lower accuracy. 

In this paper, we propose a new PR system enhancement that uses the unexploited factor of adaptively increasing the time window size when required. To the best of our knowledge, no framework based on the adaptive windowing of EMG signal has been proposed in the literature. The enhancement is simple in the sense that it is based on thresholding the output likelihood of the classifier to check the confidence in the decision. This provides an improvement in the classification performance at the expense of a very small fraction of extended time windows. The proposed adaptive window size framework is only used for low confidence decisions. The underlying hypothesis is that the system will adapt to the difficult decisions by increasing window size until a confident classification can be achieved. The proposed novel adaptive window size framework will be validated on EMG data acquired from nine transradial amputees who performed nine classes of hand and finger movements. It will also be tested with different feature extraction methods and movement/channel combinations.

## 2. Materials and Methods 

### 2.1. Proposed Adaptive Windowing-Based PR Framework

**Figure 1:**
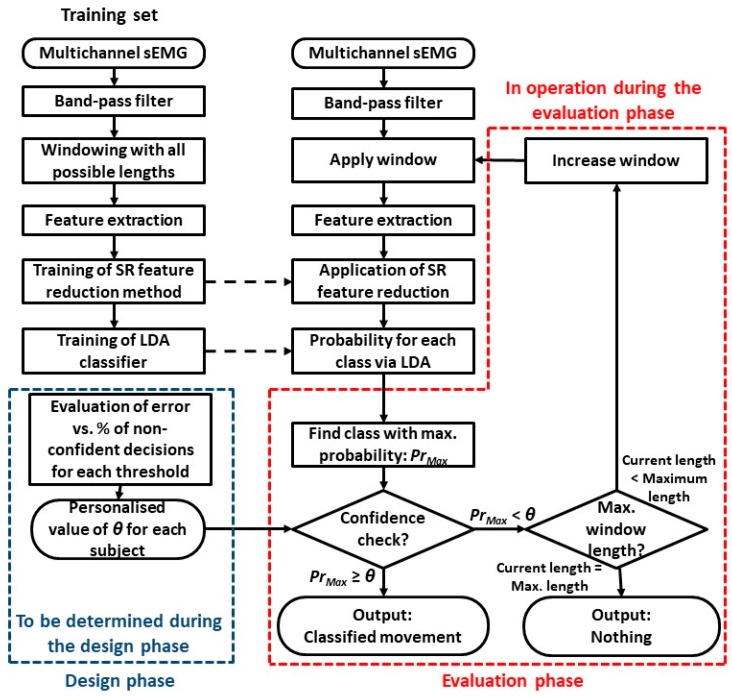
Block diagram of the proposed framework for adaptive windowing based on pattern recognition (PR).

In this section, our novel adaptive windowing-based PR framework will be presented. Figure 1 shows the block diagram of the PR system with adaptive windowing. It illustrates the main steps followed in the design and evaluation phases of the system and highlights the differences with traditional approaches by enclosing them with a dashed line (blue for the design phase and red for the evaluation one).

First of all, using the feature reduction and classification technique of choice (spectral regression and LDA in our case), the classifier is trained with features extracted from a range of window lengths. Traditionally, PR systems consider only a single window length. Instead, our adaptive windowing framework will train the classifier simultaneously with features extracted from window lengths from 150 ms to 350 ms, in steps of 50 ms. Once the classifier has been trained, it is used, without any changes, in the evaluation phase.

The key element of our system is the introduction of a threshold to evaluate if there is enough “confidence” in the result of the classification. Traditional systems output the movement with the largest probability, *Pr*, no matter how large such probability is in comparison with those estimated for other classes. For example, for a three-class problem with outputs, *Pr_A_* = 0.34, *Pr_B_* = 0.33 and *Pr_C_* = 0.33, for classes A, B and C, respectively, the traditional approach will decide on class A even though the output for class A is very similar to those of B and C. Instead, we introduce a threshold (*θ*). For each window, the largest *Pr* among all classes (*Pr_Max_*) is compared against *θ*. If *Pr_Max_* ≥ *θ*, we consider that there is enough confidence in the decision and the system outputs the class associated with *Pr_Max_*. Instead, if *Pr_Max_* < *θ*, we consider that the system does not have enough confidence in the decision and the system will increase the window size. The features will be recomputed for the increased window size and the classification will be reattempted with the expectation that the classifier will now provide a more confident decision since a large window size may have less variability in the extracted features [24]. This iterative process is repeated until *Pr_Max_* ≥ *θ*, in which case the system outputs the corresponding class, or the maximal window length has been reached, in which case the system does not produce any output. In this way, the system implicitly refuses to output a class when the classification confidence is not high enough, in a similar way to [29].

A crucial parameter of the system is the value of *θ*. We considered 16 values in *θ* = {0.70, 072, 0.74, …, 0.96, 0.98, 0.99}. *θ* is fine-tuned individually for each subject in the design phase, when the system assumes a fixed value of *θ* and estimates the performance of the system in terms of percentage of erroneous classifications and the percentage of increased windows, which depends on how many windows needed to be extended. This evaluation is then repeated for all possible values of *θ*. The results can be visualized as performance curves. Lower *θ*s imply that a lower level of confidence is required to accept a classification decision. This leads to faster decisions (shorter windows) at the expense of more errors. In contrast, higher *θ*s tend to be associated with lower error rates but larger windows. A trade-off balancing both factors is achieved by carrying out a visual inspection of the curves. The value of *θ* chosen for each subject will be utilized in the evaluation phase without any modification.

### 2.2. Data Collection

Nine amputees (seven acquired males and two congenital females) with unilateral amputation were recruited with average age 31.8 ± 10.6 years (mean ± standard deviation, SD). Time since amputation was 7.6 ± 8.8 years (mean ± standard deviation, SD). The demographic information of the amputees is illustrated in Table 1. For TR1-TR6 amputees, the EMG datasets were collected at Al-Muthana Rehabilitation Centre for the Iraqi Army (Baghdad) and Babylon Rehabilitation Centre, Iraq, while for TR7 (Transradial 7), CG1-CG2 (Congenital 1 & 2), the EMG datasets were collected at Plymouth University, UK. The study was conducted in accordance with the Declaration of Helsinki and the protocol was approved by the Ethical Committee at the School of Computing and Mathematics, Plymouth University (17 September 2009) and updated to collect the data from Iraq. Written informed consent was approved by amputees to join the study.

**Figure 2:**
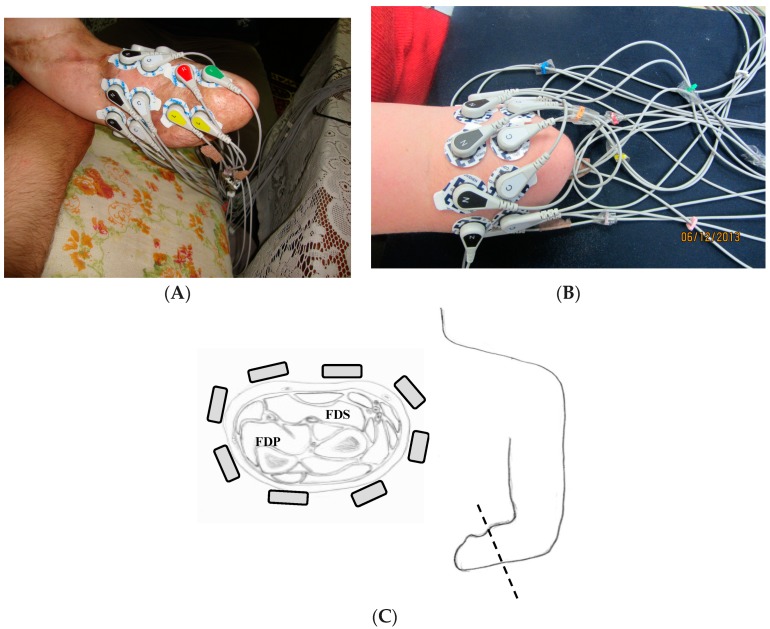
Examples of electrode locations for 2 amputees, (**A**) Transradial amputee TR4, (**B**) Congenital amputee CG2, (**C**) Cross section of the forearm with approximate electrode locations over the muscles: Flexor Digitorum Superficialis (FDS) and Flexor Digitorum Profundus (FDP) for Transradial amputee TR4.

At the beginning, participants’ skin was cleaned with alcohol. Afterwards, abrasive skin preparation gel (NuPrep^®^, D.O. Waver and Company) was put on the skin. Seven pairs of Ag/AgCl electrodes (Tyco healthcare, Ratingen, Germany) were utilized and placed around the left stump in one or two rows arrangement for all amputees. The exception was amputee CG2 in which the electrodes were put on the right stump. The reference electrode was placed on the Olecranon process of the Ulna. Figure 2 shows examples of the electrode locations for 2 amputees, TR4 and CG2. It should be noted that the electrode locations for each amputee was variable as each amputee has different stump morphology. 

The EMG signals were acquired with custom-build pre-amplifier. The electronic system of the preamplifier consisted of a 2-stage amplifier (gain factor of 1000). The preamplifier has also two analogue filters to reduce noise, low-pass filter (fourth-order Butterworth, *f_c_* = 450 Hz) and high-pass filter (second-order Butterworth, *f_c_* = 10 Hz). Then, NI USB 6210 (National Instruments, Austin, TX, USA) was utilized for analogue to digital conversion (sampling frequency = 2000 Hz). LabVIEW software package (National Instruments, USA) was utilized to develop a virtual Instrument (VI) for signal acquisition and display. 

**Figure 3:**
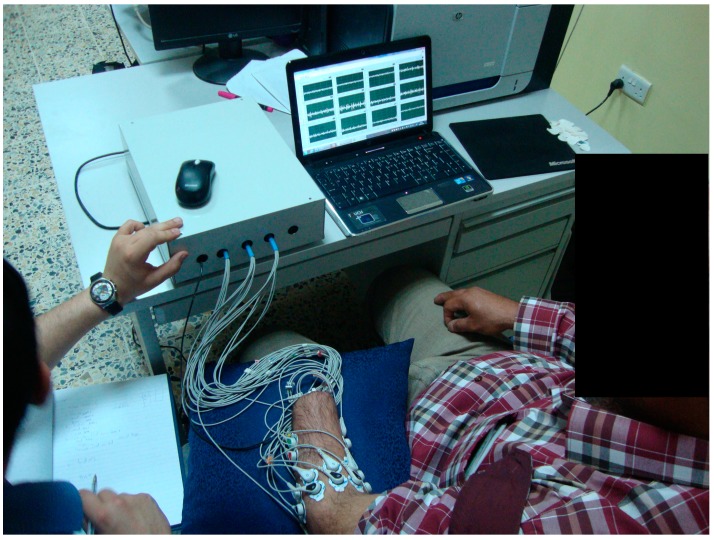
Picture showing the protocol used in this study where amputee TR5 is using the intact-hand to help him imagine the fine pinch movement with his stump.

EMG signals were acquired for nine classes of movements, which are: (1) Thumb flexion; (2) Index flexion; (3) No movement (rest); (4) Fine pinch; (5) Tripod grip; (6) Hook grip; (7) Spherical grip; (8) Pronation and (9) Supination. For each of the movements, the amputees produced constant, non-fatiguing contraction with moderate force level. In order to help them to imagine the needed movement, the amputees used their intact-hand and/or visual feedback from the raw EMG signals. Figure 3 shows amputee TR5 performing the protocol of imaging the fine pinch movement with the help of the intact hand. We recorded five to eight trials for each movement class, for each amputee. A trial represents a holding phase of 8–12 s. 

### 2.3. Investigation of the Effect of Different Feature Extraction Methods on the Adaptive Windowing Framework

In order to evaluate the proposed PR windowing framework based on adaptive window size, the EMG signals were split into three sets, (a) training (2 trials), (b) validation (2 trials) and (c) testing (rest of the trials, 1 to 4 trials). The training and validation sets (Figure 1) were used to find the trade-off threshold for each amputee (*design phase*), while the unseen testing set was utilized to evaluate the final performance (*evaluation phase*) as will be explained next and shown in the results section. For more information about the details of the design and evaluation phases, the reader is refereed to Table 2.

#### 2.3.1. Design Phase

In the *design phase*, the training and validations sets were used to find the trade-off threshold for each amputee. First, the EMG signal of the *training set* (Table 2) is band pass filtered 20–450 Hz. Three feature extractions were used to investigate the suitability of our proposed framework with different feature extraction methods. These methods include (1) Time Domain-Power Spectral Descriptors (TD-PSD) [3]; (2) Wavelet features [3], where the energy of the coefficients at each node were calculated with 5 levels decomposition and Symmlet-8 family; and (3) Time-domain features (TD) [30] including: integral absolute value, waveform length, number slope sign changes and the number of zero crossings.

We extracted these features for 150 ms window size and an increment of 50 ms. In order to train the classifier with features of multiple window sizes, we also extracted these features for other window sizes involved in the adaptive window size algorithm that is, 200, 250, 300 and 350 ms. The features from all window sizes were combined in the training data. To reduce the dimensionality of the extracted features, the Spectral Regression (SR) dimensionality reduction method was used [31,32]. With SR, mapping of the feature set is done into a new reduced domain with the size of *c* − 1 features only, where *c* is the number of movement classes.

**Figure 4:**
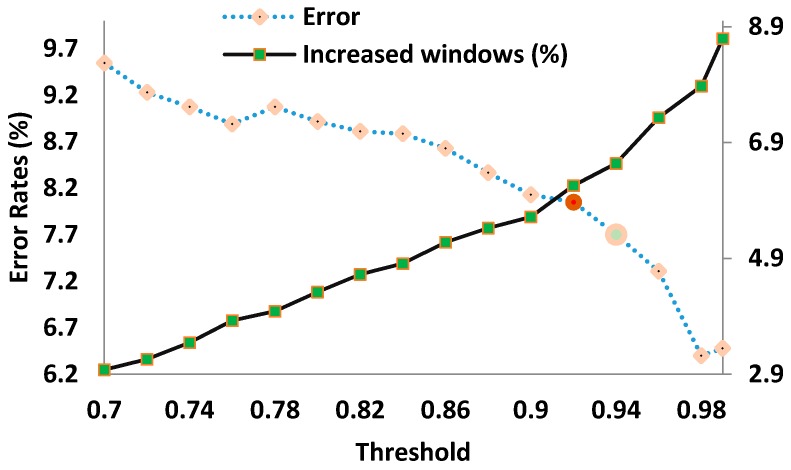
An example of the behaviour of the error of classification (on the validation set—see Table 2) and percentage of increased windows with the classifier trained with the *training set* and tested with the *validation set* for the case of 9-movement classes with 7 electromyogram (EMG) channels for amputee TR6 with TD-PSD feature extraction method. Primary axis is the error rates (dotted line) and secondary axis is the % of increased windows (solid line). The trade-off threshold is shown as a red circle.

An LDA classifier with output probabilities was used to perform classification [11,30]. The LDA classifier in the design phase is trained with reduced features from all window sizes and tested with EMG features from the validation set and the adaptive windowing framework. The algorithm was tested with 16 different threshold values for each amputee {0.7, 0.72, 0.74, 0.76, ..., 0.94, 0.96, 0.98 and 0.99}. For each subject, we calculated the classification errors (Equation 1) (for the validation set) and the percentage of windows larger than 150 ms (% of increased windows) for each threshold value. For all threshold values, we plotted a single graph containing the error rates plotted on the primary axis and the percentage of increased windows plotted on the secondary axis. The axes range were set to the minimum and maximum values of the error rates and percentage of increased windows for each individual amputee. The threshold value that is located after intersection point of the two curves was chosen as the trade-off threshold for that amputee. Figure 4 shows an example of the process of finding the trade-off threshold for TR6 amputee for the case of 9-movement classes with 7 EMG channels (TD-PSD FE method was used here) where the chosen trade-off threshold value was 0.92. It should be noted that the *design phase* was only utilized to obtain the trade-off threshold value for each individual amputee. 

The classification error rates will be calculated according to [30] as follows

(1) $$\;Classification\;error\;rate\;\left(\%\right)=\;\frac{Number\;of\;incorrect\;decisions}{Total\;number\;of\;decisions}\;\times \;100\;$$

#### 2.3.2. Evaluation Phase

In the *evaluation phase*, we utilize the testing set (Table 2) to evaluate the performance of the proposed adaptive windowing method for each amputee with the trade-off threshold calculated in the previous phase (*design phase*) with the use of the same PR chain that is, the same feature extraction (TD-PSD, WT and TD), SR and adaptive windowing. The classification error rates were calculated for all FE methods (Equation (1)).

We utilised a Bonferroni-corrected Analysis of Variance (ANOVA) test with a significance level of 0.05 to test the statistical significance of the achieved classification results, where the null hypothesis is that the errors of classification obtained by the proposed adaptive windowing framework, for each feature extraction set (i.e., TD-PSD, Wavelet and TD) are not significantly different from each other. The null hypothesis will be doubted if the *p-*values are small (<0.05), which may infer that the performance of the different methods is significantly different.

### 2.4. Investigation of the Effect of Different Movement/ Channel Combinations on the Adaptive Windowing Framework

In order to evaluate the effectiveness of the proposed adaptive windowing-based PR framework, different number of movement classes (7 and 9 classes) with 2 EMG channel sets (5 and 7 channels) were investigated with the proposed adaptive windowing framework, LDA classifier and the best performer for the feature extraction methods from the previous analysis in Section 2.3.2. For the case of 7 movement classes, the first 7 movements from all 9 movements were chosen, that is, (1) Thumb flexion; (2) Index flexion; (3) Fine pinch; (4) No movement (5) Tripod grip; (6) Hook grip; (7) Spherical grip (see Section 2.2). As for the case of 5 EMG channel, the first 5 EMG channels were chosen from the full set of 7 EMG channels.

Bonferroni-corrected Analysis of Variance (ANOVA) tests with a significance level of 0.05 were utilized to test the statistical significance of the achieved classification results, where the null hypothesis is that the errors of classification obtained by the proposed adaptive windowing framework with different EMG channel combinations are not significantly different from each other. In such a situation, the performance of each of the two different channel combinations is significantly different from each other, if the *p-*values are less than 0.05.

## 3. Results

### 3.1. Investigation of the Effect of Different Feature Extraction Methods

The classification error rates for the proposed adaptive windowing framework, for all nine amputees with three feature extraction methods, (a) TD-PSD, (b) Wavelet, (c) TD, for the case of for 9 movement classes with 7 EMG channels, are illustrated in Table 3. Mean and Standard Deviation (SD) are also shown in Table 3. 

**Figure 5:**
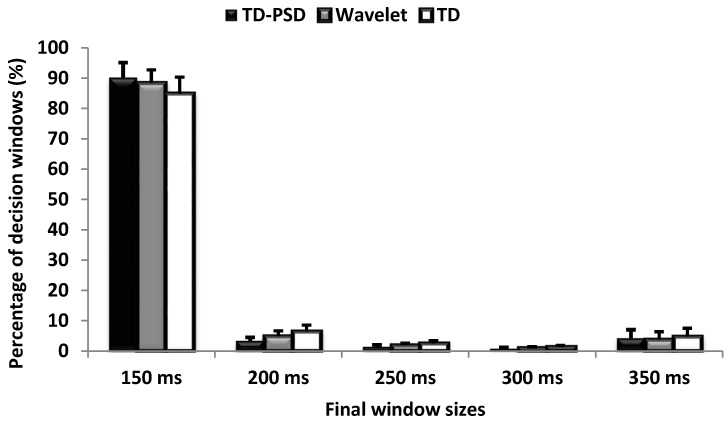
Average percentages for each of the window sizes for the case of nine movements with 7 EMG channels with 3 feature extraction methods (TD-PSD, Wavelets and TD) and the proposed adaptive windowing framework. Error bars represent SD across nine amputees.

The average percentages for each of the window sizes for the case of nine movements with 7 EMG channels with three feature extraction methods (TD-PSD, Wavelets and TD) are shown in Figure 5 where error bars represent SD across nine amputees. It should be noted that the percentage of increased windows (windows longer than 150 ms) was higher in TD and Wavelet than in TD-PSD. Only around of 10% of all windows with TD-PSD FE have undergone an increase in the window size by the proposed adaptive windowing framework while the rest of windows led to probabilities above the threshold (Figure 1).

The results of statistical tests (Bonferroni corrected *p*-values) showed that there are no significant differences between each pair of the 3 feature extraction methods as *p*-values > 0.05.

### 3.2. The Effect of Different Movement/Channel Combinations

**Figure 6:**
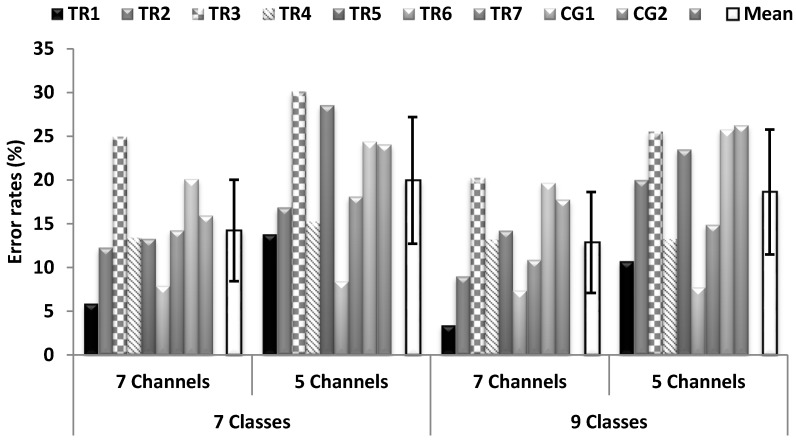
Error rates and the mean over 9 amputees for the proposed adaptive windowing framework and TD-PSD feature extraction (FE) method, with different movement/channel combinations for all nine amputees. SD is shown with error bars.

In this section, the best feature extraction method from the previous analysis (TD-PSD) will be used to investigate the effect of using different movement/channel combinations. Figure 6 displays the results of testing the proposed windowing framework with the trade-off threshold values, for the 7- and 9-movement-class problems and for each of five and seven EMG channel cases for all nine amputees.

In all movement/channel combinations investigated (Figure 6), the average error rates for the proposed adaptive windowing framework are within the range 13–20%. The results of statistical tests showed that for the cases of 7 and 9 movement classes, there are significant difference between the 5 and 7 EMG channels (*p*-values < 0.05).

Table 4 illustrated the average final window size ± SD across all amputees for all cases of movement/channel combinations investigated. This was calculated by taking the average of the final window size for all windows in the test set for each amputee. Clearly, it can be seen that as the number of EMG channels were reduced, the average window size increases since the classification problem is more difficult with only 5 channels than with 7 EMG channels.

In Table 5, the trade-off threshold values (*θ*) for each amputee are shown for the cases of 9-movement classes with 5 and 7 EMG channels.

Table 6 presents the results of the classification accuracy for each of the 9 movement classes with TD-PSD FE and the proposed adaptive windowing framework with 5 and 7 EMG channels. 

## 4. Discussion

We proposed an adaptive windowing framework which increases the window size of the EMG signal to acquire more information for difficult decisions whose confidence lies below a certain threshold value (*θ*). The proposed framework was tested with three different FE methods, namely, TD-PSD, WT and TD. The proposed adaptive windowing framework was also validated with different combinations of movement classes and number of EMG channels. 

LDA was chosen to be used with our proposed framework since it has shown good performance for PR systems and it has few tuneable parameters [3,33]. The proposed framework can be extended to work with other classifiers that produce output probabilities.

In our proposed framework, we utilize the information from the EMG signal to improve the performance at the expense of potentially increasing the window size. On one hand, this increase in the window size only happens for the suspicious decisions with low confidence after implementing a confidence checker for the LDA classifier. On the other hand, increasing the window size can reduce the variability within the EMG features [24]. The decision of rejecting a window is based on the ground of safety. Otherwise, the class with the highest probabilities will be chosen as the output class (Figure 1).

For comparative purposes, Table 7 presents the average classification errors for the case of 9 movement classes with 5- and 7- EMG channels with TD FE and the classical LDA, LDA with MV across the current and 8 previous decisions [9], LDA with BF also across the current and 8 previous decisions, in a similar manner to [10] and our proposed adaptive windowing framework. It can be seen that the proposed adaptive procedure manages to decrease the error rates and outperforms other methods. 

In Table 2, we showed that our work can still be applied with different FE methods, that is, TD-PSD, Wavelet transform and TD. The error rates of TD-PSD (13%) were lower than that of TD and WT. Unlike [11,13,24] who used one FE which was TD, we utilised three other FE methods in this study. It is noteworthy to mention that TD-PSD outperformed TD FE method, in agreement with the recently published literature [34,35].

In Figure 6, it should be noted that the error rates for 7 movement classes with 5 channels were higher than for 9 movement classes with 5 EMG channels. This may be because the errors appeared mainly in the second movement which is index flexion movement (within the first 7 movement classes). However, the errors in pronation and supination grips (in the set of 9 classes) were much less than that of the index flexion (see Table 6). This may be a potential reason for why 7 classes have larger errors than 9 classes. 

The errors shown in Figure 6 are still in the range of 13–20% and away from that of a usable system [30]. However, our adaptive windowing method is based on the EMG signal itself and it is applicable to most EMG control strategies. We illustrated our proposed windowing framework with LDA in challenging classification tasks carried out by nine amputees and we expect it to be able to improve the performance of other PR-based systems. The recent deployment of a PR system in commercial prostheses shows the importance of such systems for the control of upper- limb hand prostheses [4]. 

In Table 4, the average of the final window size obtained during the evaluation phase (Section 2.3.2) was illustrated for all nine amputees. We acknowledge that our method results in an increased window size for a fraction of movement instances. However, we argue that this increase in the window size is not fixed for all windows and it is only for particular windows of the signal where a difficult decision is identified, that is, when the output probability of the classifier is below a certain threshold (*θ*). Our windowing framework only increases the window size to get the final output for the difficult decisions which is a small fraction of the total decisions (around 10–15% for the case of 9-movement classes with 7 channels). Among these 10–15%, only a small fraction reached the maximum window size of 350 ms as shown in Figure 5. 

Unlike the work presented in [13] where a fixed threshold value was used for all subjects, it is shown in Table 5 that the threshold value (*θ*) is different for each amputee and subject-specific tuning of the threshold value of the algorithm is essential. We also showed an example of finding the threshold value (*θ*) for 1 amputee in Figure 4. It is worth noting that normal subjects recruited in [13] have less variance in their performance while, in our study, we recruited nine transradial amputees where each amputee has different stump morphology and time since amputation. 

In our proposed windowing framework, only uncertain decisions are corrected. The limitation of the simple movement rejection approach was acknowledged in [36], where the unintended activation of a device was also suggested to be a potential reason for frustration while testing pattern recognition systems [19]. It should be noted that the approach proposed in [11] has many tuneable parameters unlike our proposed framework where only the threshold (*θ*) needs to be adjusted. 

Table 6 illustrated the classification accuracy for each class, for all nine amputees, for the case of 9 movements classes with 5 and 7 EMG channels, where most of the errors were in the index flexion movement. As expected, the rest movement was the class with the lowest errors. 

We started with a window size of 150 ms and adopted an increase in the window size of 50 ms, that is, 200, 250, 300 and 350 ms, with 50 ms window increment. Larger steps with steps of window size can be used such as 100 ms to reduce the iterations of the algorithm and to help real-time implementations. Nonetheless, it must be noted that the key element in our windowing framework is the estimation of a personalised threshold (*θ*) in the design phase (Section 2.3.1). This forms part of the training of the prosthesis. However, once *θ* has been set, the system could operate in real-time. It might be advisable, though, to readjust the value of *θ* periodically to accommodate slow changes in the patterns of the EMG signals due to training in the use of the prostheses, for example. To calculate the error rates, the classification errors were computed without including the rejected windows, which account for only a very small fraction of the results.

Finally, offline analysis was performed in this study which may a limitation, similar to other studies [11,13]. In addition, a relatively small number of movement classes was collected in this study. Future studies will collect more movement classes from more amputees and consider regression approaches. We note, however, that our framework is applicable as well to regression-based prosthesis control.

## 5. Conclusions

A novel adaptive windowing framework was proposed as an enhancement for the PR systems. The proposed novel framework was validated with LDA classifier and our proposed concept can be applied to other classifiers. The proposed approach estimated the output confidence of each individual movement classification process and increased the window size only if a decision with a probability below a certain threshold value (*θ*) is detected. The proposed adaptive windowing framework was tested with EMG datasets collected from nine transradial amputees who performed nine movement classes. Classification error rate of 13 % was obtained with TD-PSD and the proposed adaptive framework for 9 movements classes. Recent deployment of PR systems on commercial prostheses gives hope to improve the existing prosthesis control. The PR enhanced system proposed in this study is a further step towards improving the control of the upper limb prostheses.

## Figures and Tables

**Table 1:** The details of the demographic information of the transradial amputees recruited in this study.

ID	Age (y)	Sex	Stump Length (cm)	Time Since Amputation	Type of Prosthesis
TR1	25	M	13	4 years	Cosmetic
TR2	33	M	18	6 years	None
TR3	30	M	29	28 years	Cosmetic
TR4	27	M	16	4 years	Body powered
TR5	35	M	23	8 years	Cosmetic
TR6	29	M	24	7 years	Cosmetic
TR7	57	M	14	3 years	None
CG1	19	F	9	N/A	Myoelectric 3–12 y
CG2	31	F	10.5	N/A	Myoelectric 8–16 y

**Table 2:** Summary of the details of the design and evaluation phases.

	Design Phase	Evaluation Phase
Classifier is trained with	training set (2 trials)	training and validation sets (4 trials)
Classifier is tested with	validation set (2 trials)	unseen testing set (1 to 4 trials)
Purpose	To find the optimal threshold (*θ*)	To calculate classification errors to assess the performance of our method in an unbiased way

**Table 3:** Error rates for the proposed adaptive windowing framework (9 movements with 7 EMG channels) for all nine amputees, with 3 feature extraction methods, (a) Time Domain Power Spectral Descriptors (TD-PSD), (b) Wavelet, (c) Time Domain (TD). Mean and SD are also shown.

ID	Feature Extraction		
	TD-PSD	Wavelet	TD
TR1	3.4	7.9	9.6
TR2	9.0	13.3	20.5
TR3	20.3	21.0	16.4
TR4	13.2	11.7	11.2
TR5	14.2	17.4	15.0
TR6	7.4	9.2	8.7
TR7	10.9	11.2	9.7
CG1	19.7	18.0	17.9
CG2	17.8	17.2	25.6
Mean	12.9	14.1	14.9
SD	5.8	4.5	5.7

**Table 4:** Mean ±SD of the final window size when tested with the test set (*evaluation phase*) in ms with the standard deviation for all 9 amputees with TD-PSD FE method.

Number of Movements	Number of Channels	Average of Final Window Size (ms)
7 Movements	5 Channels	176.6 ± 14.6
	7 Channels	162.2 ± 5.8
9 Movements	5 Channels	169.7 ± 11.6
	7 Channels	162.7 ± 7.3

**Table 5:** Example of threshold values for 9 movements with 5 and 7 EMG channels for nine amputees with TD-PSD FE method.

Amputee ID	Trade-Off Threshold (*θ)* Values	
	9 Movements with 5 EMG Channels	9 Movements with 7 EMG Channels
TR1	0.92	0.96
TR2	0.88	0.96
TR3	0.92	0.94
TR4	0.94	0.92
TR5	0.96	0.96
TR6	0.92	0.92
TR7	0.82	0.84
CG1	0.88	0.98
CG2	0.74	0.74

**Table 6:** Overall average classification accuracies for each movement-class in percent (and their respective standard deviation for nine amputees), for the case of 9 movements with 5 EMG channels and for the case of 9 movements with 7 EMG channels with TD-PSD and the proposed adaptive windowing framework.

No.	Movement	9 Movements with 5 EMG Channels	9 Movements with 7 EMG Channels
1	Thumb flexion	86 ± 10	92 ± 9
2	Index flexion	68 ± 30	78 ± 31
3	Fine pinch	84 ± 27	84 ± 27
4	No Mov.	99.5 ± 0.5	99 ± 1
5	Tripod grip	82 ± 28	91 ± 12
6	Hook grip	83 ± 17	82 ± 20
7	Spherical grip	68 ± 17	77 ± 12
8	Pronation	92 ± 6	97 ± 2
9	Supination	86 ± 21	95 ± 8

**Table 7:** Mean ± SD of the classification errors for the case of 9 movement classes with 5- and 7- EMG channels with the classical LDA, LDA with MV, LDA with BF and our proposed adaptive windowing framework.

	9 Movements	
Method	7 Channels	5 Channels
Classical LDA	20.8 ± 7	28.2 ± 10.2
LDA with BF	17.3 ± 6.5	24.8 ± 10.1
LDA with MV	17.7 ± 6.4	24.9 ± 9.8
Adaptive windowing Framework	14.9 ± 5.7	23.6 ± 9.6
